# Detecting SARS-CoV-2 in the Breath of COVID-19 Patients

**DOI:** 10.3389/fmed.2021.604392

**Published:** 2021-03-17

**Authors:** Xiaoguang Li, Jing Li, Qinggang Ge, Yuguang Du, Guoqiang Li, Wei Li, Tong Zhang, Lei Tan, Runqiang Zhang, Xiaoning Yuan, He Zhang, Chen Zhang, Wenjun Liu, Wei Ding, Liang Sun, Ke Chen, Zhuo Wang, Ning Shen, Jun Lu

**Affiliations:** ^1^Department of Infectious Diseases, Peking University Third Hospital, Beijing, China; ^2^CAS Key Laboratory of Pathogenic Microbiology and Immunology, Institute of Microbiology, Chinese Academy of Sciences, Beijing, China; ^3^State Key Laboratory of Biochemical Engineering, Institute of Process Engineering and Innovation Academy for Green Manufacture, Chinese Academy of Sciences, Beijing, China; ^4^Commune of Scientific Engineers, Institute of Physics, Chinese Academy of Sciences, Beijing, China; ^5^State Key Laboratory of Stem Cell and Reproductive Biology, Institute of Zoology, Chinese Academy of Sciences, Beijing, China; ^6^Department of Infectious Diseases, YouAn Hospital, Capital Medical University, Beijing, China; ^7^State Key Laboratory of Hydroscience and Engineering, Department of Energy and Power Engineering, Tsinghua University, Beijing, China

**Keywords:** SARS-CoV-2, COVID-19, exhaled breath, swirling aerosol collector, virus detection

## Abstract

In the COVID-19 outbreak year 2020, a consensus was reached on the fact that SARS-CoV-2 spreads through aerosols. However, finding an efficient method to detect viruses in aerosols to monitor the risk of similar infections and enact effective control remains a great challenge. Our study aimed to build a swirling aerosol collection (SAC) device to collect viral particles in exhaled breath and subsequently detect SARS-CoV-2 using reverse transcription polymerase chain reaction (RT-PCR). Laboratory tests of the SAC device using aerosolized SARS-CoV-2 pseudovirus indicated that the SAC device can produce a positive result in only 10 s, with a collection distance to the source of 10 cm in a biosafety chamber, when the release rate of the pseudovirus source was 1,000,000 copies/h. Subsequent clinical trials of the device showed three positives and 14 negatives out of 27 patients in agreement with pharyngeal swabs, and 10 patients obtained opposite results, while no positive results were found in a healthy control group (*n* = 12). Based on standard curve calibration, several thousand viruses per minute were observed in the tested exhalations. Furthermore, referring to the average tidal volume data of adults, it was estimated that an exhaled SARS-CoV-2 concentration of approximately one copy/mL is detectable for COVID-19 patients. This study validates the original concept of breath detection of SARS-CoV-2 using SAC combined with RT-PCR.

## Introduction

During 2020, the human race suffered from the COVID-19 pandemic (1–6) but fortunately learned the importance of controlling transmission through air (7–11). As a main source of aerosol spread of respiratory viruses, exhaled breath of patients has been confirmed as a potential risk using various methods of sampling and subsequent testing (12–25). Several groups have reported SARS-CoV-2 positivity in samples of exhaled breath condensate (EBC) (21–25), and based on Ct values, Ma et al. further showed that EBC-positive COVID-19 patients exhaled millions of SARS-CoV-2 RNA copies per hour (23). Standard diagnosis of viruses based on swabs or serum has been rapidly developed (17, 26–29), while auxiliary methods, such as trace biomarkers of COVID-19 in exhalations, have also been explored (18–20). Nevertheless, there is an urgent need for novel methods to detect SARS-CoV-2 in the transmission path.

**Figure 1:**
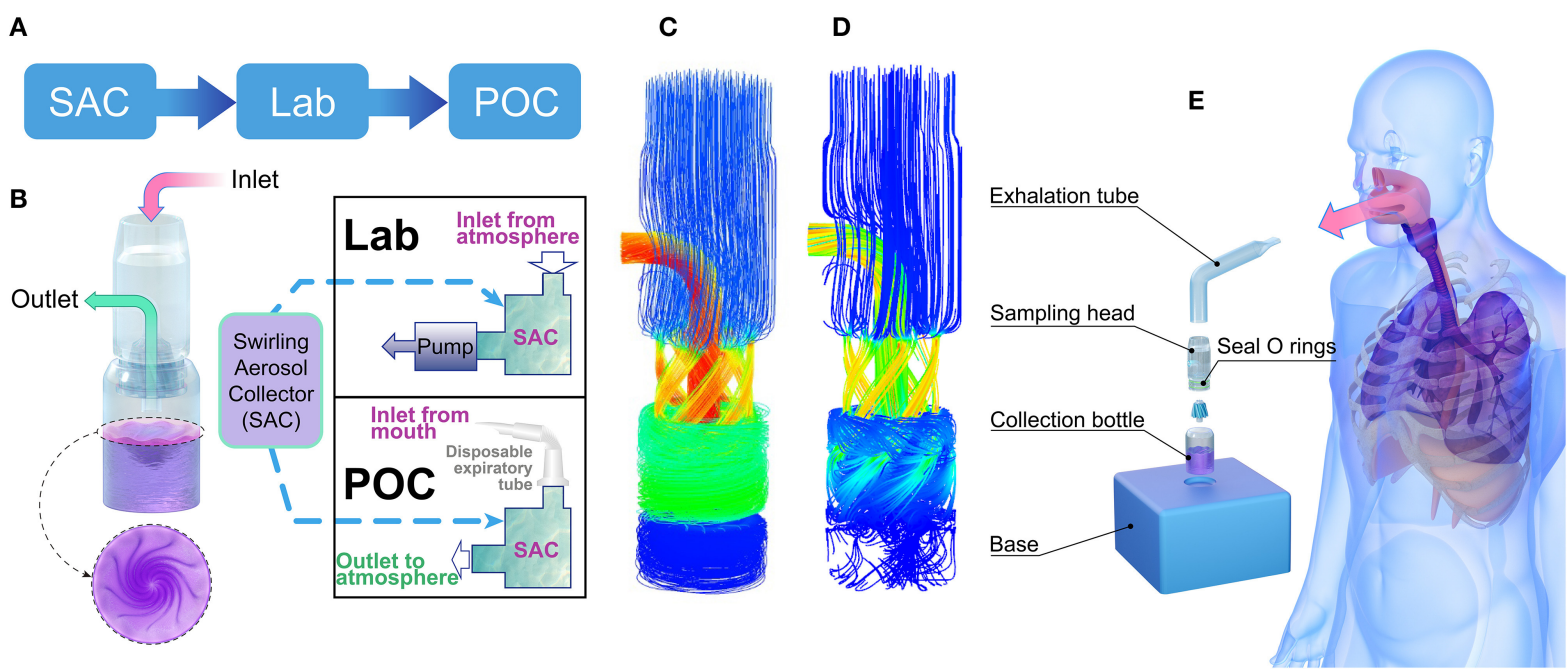
Aerosol-collecting device and process. **(A)** Project flowchart. **(B)** Structure of the swirling aerosol collector and two modes of application: lab test mode with pump suction at the outlet and POC clinical testing mode with a disposable expiration tube at the inlet for collecting breath. **(C)** Simulated flow field demonstration using a mass flow diagram for the Lab test mode, where the inlet and outlet pressures were set to atmospheric and vacuum pressures, respectively. **(D)** Simulated flow field demonstration using a mass flow diagram for the POC testing mode where the mass flow rate at the inlet was set to 10 L/min. **(E)** Cartoon of the POC clinical testing model where instant aerosol sampling from the whole airway is demonstrated. SAC, swirling aerosol collector; Lab, laboratory evaluation; POC, point of care.

Our study aimed to develop a tool to collect viruses in exhaled breath or indoor air and rapidly determine if the source was a transmission risk of SARS-CoV-2 via aerosols, which is the route that is most challenging for epidemic control (9, 10). This work consists of three parts: the development of a swirling aerosol collection (SAC) device, laboratory evaluation (Lab), and point-of-care (POC) clinical testing (Figure 1A).

## Materials and Methods

### Collection Device

The collection device consisted of an exhalation tube, a sampling head, a collection bottle, and a base (Figure 1). The sampling head is the most important component; swirling flow channels can be rapidly 3D printed for one-time use. The cylinder bottle was made of glass with a height of 5 cm, an outer diameter of 2 cm, and a wall thickness of 0.5 mm. A double O-ring seal ensured airtightness between the collection head and the collection bottle, and the base played a role in the auxiliary fixture. The unassembled device is small enough to be carried in a pocket. The volume of the sampling solution was 2 mL and consisted of (in weight percent) 0.8% sodium chloride, 0.04% potassium chloride, 0.014% calcium chloride, 0.02% magnesium sulfate (heptahydrate), 0.012% disodium hydrogen phosphate (heptahydrate), 0.006% potassium dihydrogen phosphate, 0.035% sodium bicarbonate, 0.1% glucose, and 0.002% phenol red sodium salt.

Ansys finite element method CFX software was used to simulate the virus detection device (30). CFX software consists of three basic parts: pre-processing, solver and post-processing. In the CFX pre-treatment, the isothermal heat transfer model and renormalization group (RNG) K-ε turbulence model were used, and a scalable wall function was used to solve the physical model. According to different driving modes, the boundary conditions of the inlet and the outlet were divided into a fixed static pressure in the inlet (Lab mode, as shown in Figures 1B,C) and a fixed mass flow rate (POC mode, as shown in Figures 1B,D). Smooth walls and no-slip walls were selected as wall conditions. The interface was chosen as the general connection. The advection scheme was selected as high resolution in the collection fluid, and the turbulence numerical value was selected as high resolution. The solver type of run was chosen as full, and after post-treatment, an internal streamline diagram, wall shear diagram, wall pressure diagram and middle section pressure diagram were obtained for analysis. Detailed simulation procedures and swirling aerosol behavior have also been described by Pisarev and Hoffmann (31).

### Laboratory Evaluation

All tests were carried out in a biosafety cabinet in a BSL-2 laboratory. A pseudovirus containing a synthetic RNA fragment of the SARS-CoV-2 nucleocapsid (N) gene was used to test the detection limit of the device. An Energolux air humidifier was used for ultrasonic spraying of pseudoviruses in this work. Samples with pseudovirus concentrations ranging from 10^1^ to 10^6^ pseudoviruses/mL were sprayed into a sealed 1 m^3^ biosafety chamber at a spraying rate of 100 mL pseudovirus solution per hour, with the collection device placed at the origin of the spray distance at a pumping rate of 15 L/min.

### Clinical Validation

Recruitment of COVID-19 case subjects was initiated on March 12, 2020, by two hospitals: Aid to Hubei Province National Medical Team of the Third Hospital of Peking University in Sino-French Xincheng Branch, Tongji Hospital, Huazhong University of Science & Technology (Aid-Hubei Hospital in short); and Beijing YouAn Hospital, Capital Medical University. Informed consent forms were obtained, and approval for the study was attained under Peking University Third Hospital Medical Science Research Ethics Committee 2020 (#079-02) and Ethics Committee of Beijing YouAn Hospital, Capital Medical University 2020 (#050-K). The inclusion criteria were as follows: (1) a ratio of males to females between 0.8 and 1.25; (2) each confirmed case was based on the COVID-19 protocol (6th edition, edited by the National Health Commission of China) (32); and (3) subjects voluntarily signed informed consent. The exclusion criteria were as follows: (1) subjects who were mentally incapable or unable to understand the requirements; (2) those with poor expected compliance; and (3) pregnant or lactating women.

A disposable collection device (Figure 1) was used to collect breath samples from patients over a 3–5 min sampling period in an isolated negative pressure room, and the sampling process stopped as soon as the volume of collection solution fell to 1.5 mL. RNA was extracted and tested by normal PCR in Aid-Hubei Hospital or qPCR in YouAn Hospital. Demographic data included clinical manifestations, a lung CT scan 7 d before breath sampling, a routine blood test no more than 3 d before expiratory sampling, and SARS-CoV-2 antibody testing no more than 7 d before expiratory sampling.

### Nucleic Acid Detection

RNA of collected pseudovirus was extracted and purified based on the TRIzol method using a TRIzol Plus RNA extraction kit (Thermo Fisher Scientific Co., Ltd., USA), and reverse transcription was performed with a standard method using a reverse transcription kit (Shanghai Promax Bio Products Co., Ltd.) (33). The pseudovirus containing a synthetic RNA fragment of the SARS-CoV-2 N gene was obtained from Zhishan Biotechnology Co., Ltd. (Xiamen, China). For laboratory experiments with pseudoviruses, regular PCR and qPCR experiments were both performed. The primers used in the analysis included the primer N-F (5′-GGGGAACTTCTCCTGCTAGAAT-3′) and SARS-CoV-2 N gene reverse primer N-R (5′-CAGACATTTTGCTCTCAAGCTG-3′). All primers were synthesized by Biotechnology Co., Ltd. (Shanghai). qPCR experiments were performed using an ABI Prism 7000 instrument (Applied Biosystem, Foster City, USA) according to the manufacturer's instructions. Each reaction contained 10 μL of SYBR Green Master Mix (Thermo Fisher Scientific Co., Ltd., USA), 1 μL of primer N-F, 1 μL of primer N-R, 4 μL of nuclease-free water (Thermo Fisher Scientific Co., Ltd., USA) and 4 μL of reverse transcription cDNA products. PCR experiments were performed with 12.5 μL of TaqMan Mix (2X), 1 μL of primer N-F, 1 μL of primer N-R, 7.5 μL of nuclease-free water, and 3 μL of reverse transcription cDNA products. The amplification program for both qPCR and PCR consisted of 2 min at 50°C, 2 min at 95°C, 50 cycles of 15 s at 95°C alternating with 15 s at 58°C, and 1 min at 72°C. PCR products were subjected to electrophoresis on a 1.5% agarose gel.

For experiments with clinical swab and breath samples, according to the manual of the DP315-R extraction kit (Tiangen Biotech, Beijing, China), a 200 μL sample of the collection solution was used to extract RNA. Extracted RNA was finally redissolved in a 60 μL solution of RNase-free ddH_2_O. Then, 10 μL of RNA was taken for a subsequent one-step reverse transcription and amplification reaction using BGI RT-PCR kits (BGI, Wuhan, China) targeting the SARS-CoV-2 *orf1ab* gene to detect SARS-CoV-2. The amplification program consisted of 20 min at 50°C, 10 min at 95°C, and 40 cycles of 15 s at 95°C alternating with 30 s at 60°C. Positive results were determined when the Ct value was no larger than 38, according to the kit instructions.

For positive clinical breath samples, further standard curve qPCR was performed to analyse the virus concentration. RNA extraction and one-step reverse transcription amplification were performed as described above. Standard curves were generated by diluting RNA transcribed from a SARS-CoV-2 *orf1a* gene fragment. The *orf1a* gene fragments were synthesized based on the sequence of the *orf1a* gene of WH04|2020-01-05 and cloned into a pGEM-T Easy vector (Promega Shanghai, Shanghai, China). The pGEM-orf1a vector was then linearized and used as the template for *in vitro* RNA transcription using the RiboMax Express Large-Scale RNA Production System (Promega, Madison, Wisconsin, USA). Template DNA was then digested, and RNA products were purified with an RNeasy kit (Qiagen GmbH, Hilden, Germany). The purified RNA was quantified spectrophotometrically at 260 nm. Ten-fold serial dilutions of the purified RNA were subjected to RT-PCR analyses for standard curve experiments according to the manufacturer's instructions of the ABI Prism 7000 instrument.

### Statistical Analysis

Statistical analyses were conducted with SPSS version 17.0 (Chicago: SPSS Inc.). Discrete data are presented as counts and percentages. Continuous variables are described by means and standard deviations if they were normally distributed; otherwise, variables are represented by medians and interquartile ranges.

## Results

### Simulation and Testing of the Swirling Aerosol Breath Collector

The collection device combines cyclone centrifugation and an impact collector, as designed by Willeke et al. (34). As shown in Figure 1B, the core of the collector is the cyclone guide channel, which generates centrifugal dispersion and impact force, spreading the virus into the collection liquid. The swirling process is driven by a pressure difference between the inlet and outlet. The virus particles accumulated in the collection liquid, and the enriched solution was subsequently analysed for SARS-CoV-2 nucleic acid using RT-PCR. In this study, laboratory verification and clinical trial validation tests were conducted. The collector can be run in two fluid field modes, a suction mode with an air pump at the outlet (Figure 1C) or a POC mode without the pump (Figure 1D). Simulation results of the fluid field modes showed no significant difference in collection efficiency between the two modes for particles with a diameter above 100 nm (95% in both modes). In clinical trials, the POC mode was used, with the air inlet connected to the subject's mouth (Figure 1E).

### Testing of the Collection Device Using a SARS-CoV-2 Pseudovirus

**Figure 2:**
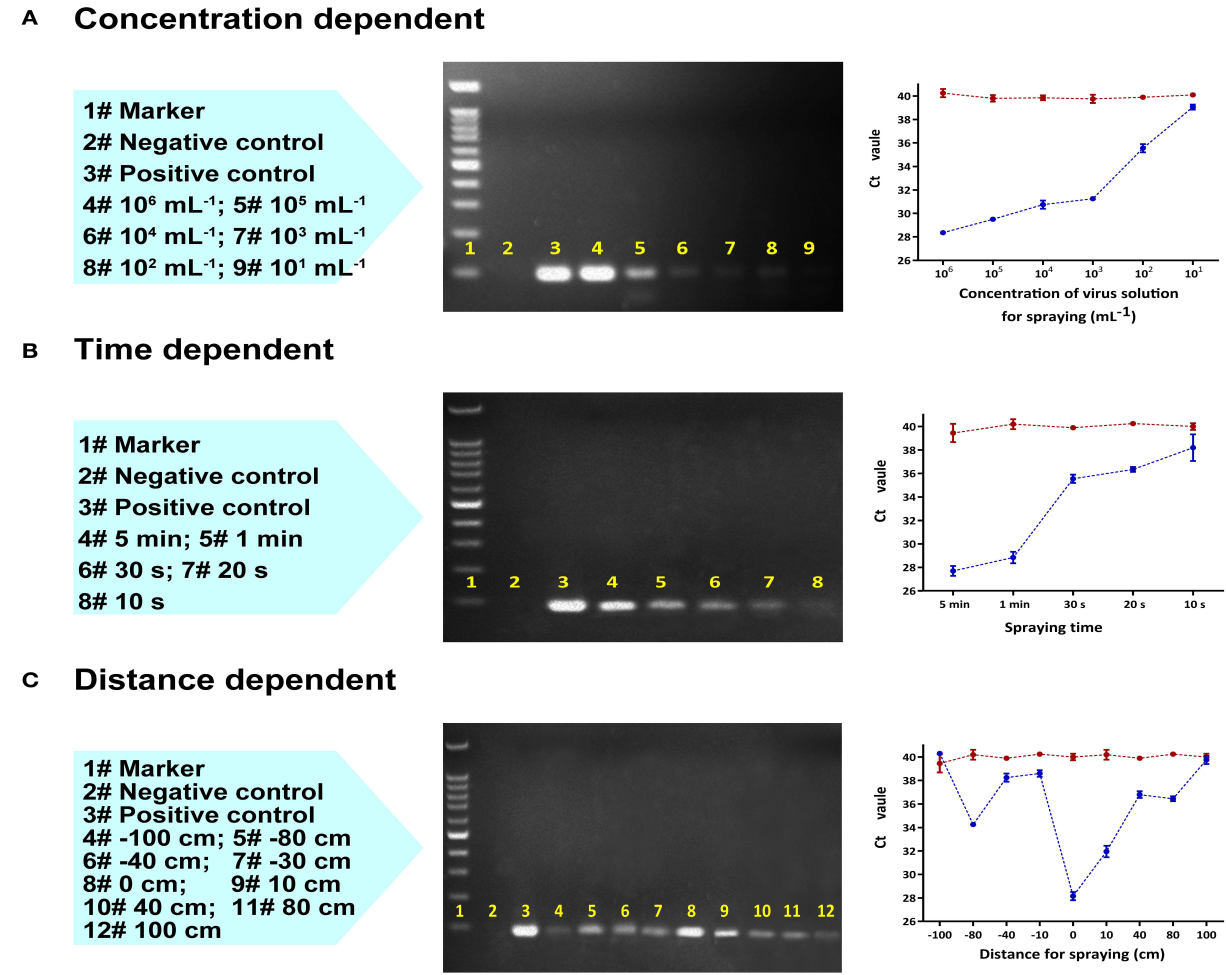
Laboratory RT-qPCR and PCR validation of the collection device using an aerosolized SARS-CoV-2 pseudovirus varying **(A)** virus concentration (mL^−1^) with a fixed sampling distance of 10 cm, **(B)** collection time (s) with a fixed sampling distance of 10 cm, and **(C)** distance between the collector and the spraying head with a fixed sampling time of 2 min and fixed concentration of 100,000 copies/mL. Left to right: Lane content description, gel image of PCR products, and RT-qPCR plot of Ct value vs. variable (the red line represents the control group, the blue line is the test group).

After 30 min of continuous collection with a sampling distance of 10 cm, the limit of detection was established at a sample concentration of 10 pseudoviruses/mL (Figure 2A). Shorter collection times were also found to be viable. At a sample concentration of 10^4^ pseudoviruses/mL, as little as 10 s was sufficient to obtain a positive result (Figure 2B).

The collection efficiency gradually decreased as the distance between the origin of the spray and the collection device was increased to 80 cm; beyond this distance, the collector could no longer effectively capture pseudovirus (Figure 2C). Collection performance was range dependent whether the aerosol sprayer was oriented towards (positive distance) or away from the collector (negative distance). In both cases, the optimal collection distance was between 0 cm and 10 cm.

### Validation by Clinical Trials

**Figure 3:**
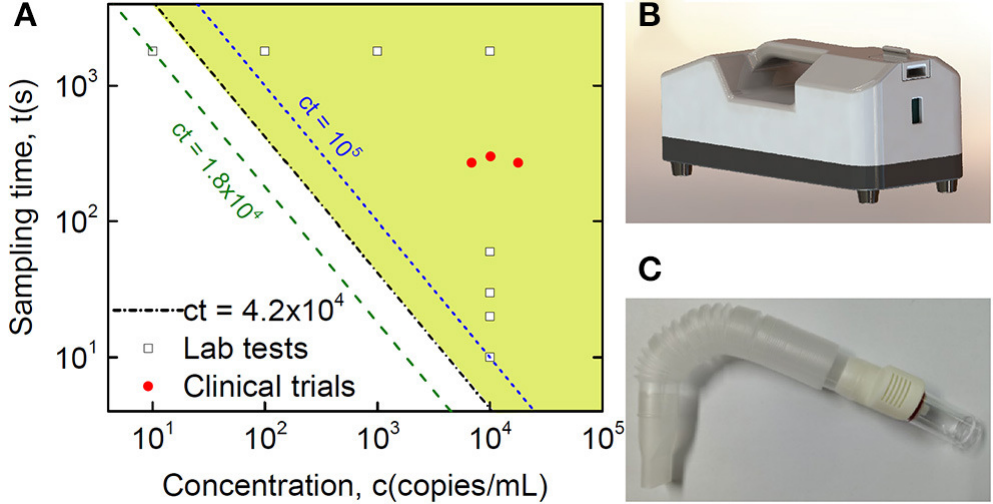
Clinical validation of SARS-CoV-2 detection in breath samples with comparison to Lab testing. **(A)** Quantitative plot of sampling time vs. virus concentration from detection tests of the Lab and collected clinical samples, where the Lab samples are sampling time-dependent tests with a fixed virus concentration at 10,000 copies/mL and concentration-dependent tests with a fixed sampling time of half an hour, while the shaded area indicates the detection range of the current method based on Lab tests, with the equation of the threshold boundary line ct = 42,000 (s·copies/mL), midway between the 18,000 and 100,000 lines. **(B)** Lab sampling device with a pump installed inside. **(C)** Disposable collection tube applied in the real POC clinical test.

Breath samples were collected from 12 healthy volunteers and 27 patients known to have COVID-19 using a disposable collection tube (Figure 3C). Nine of the patients had their breath collected twice, for a total of 48 tests (Table 1). In the healthy control group (Nos. 28–39 in Table 1), no false positives were found after parallel testing of throat swab and breath samples. All patients were originally diagnosed by positive throat swab nucleic acid tests, chest CT scans showing pneumonia, and clinical symptoms consistent with the disease. The patients included 15 males and 12 females with ages ranging from 27 to 77 years old (mean: 58 years) and courses of disease ranging from 23 to 70 d. Only four patients exhibited symptoms on the day of sampling, such as cough, expectoration, and shortness of breath after walking activity. Chest CT scans were repeated every 1–2 weeks from the beginning of hospitalization and showed ground-glass opacities in all patients' lungs. SARS-CoV-2 RT-PCR nucleic acid tests of extracted breath samples were positive in 3 cases and negative in 24 cases. Throat swab samples were positive in 10 cases and negative in 17 cases. Antibody IgM testing was positive in 21 cases and negative in 6 cases, whereas all 27 cases tested positive for IgG antibodies.

**Table 1:** Demographic, antibody, and nucleic acid detection characteristics of 27 patients infected with SARS-CoV-2.

**No**.	**Age range**	**Course of disease**^**a**^	**Symptoms**	**IgM**	**IgG**	**Swab PCR**	**Breath PCR**
1^*^	60–65	42/46	–	+	+	–	–
2^*^	50–55	29/33	–	+	+	+	–
3	70–75	47	Cough, shortness of breath after activity	+	+	–	–
4	70–75	52	–	–	+	+	–
5^*^	60–65	51/55	–	+	+	+	–
6^*^	70–75	52/56	–	–	+	–	–
7^*^	40–45	23/27	–	+	+	+	–
8^*^	25–30	45/49	–	+	+	+	–
9^*^	60–65	49/53	–	–	+	–	–
10^*^	75–80	52/56	Mild shortness of breath after activity	–	+	–	–
11^*^	55–60	54/58	Mild cough, shortness of breath after activity	–	+	–	–
12	60–65	28	–	+	+	+	–
13	40–45	56	–	+	+	+	–
14	65–70	54	–	+	+	–	–
15	65–70	45	–	+	+	–	–
16	60–65	47	–	+	+	–	–
17	70–75	56	–	+	+	–	–
18	50–55	48	–	+	+	–	–
19	40–45	70	–	+	+	–	–
20	40–45	48	–	+	+	–	–
21	50–55	55	–	+	+	–	–
22	70–75	55	–	+	+	–	–
23	50–55	46	–	+	+	–	–
24	40–45	33	–	+	+	–	–
25	50–55	36	Cough, expectoration	+	+	+	+
26	60–65	52	–	+	+	+	+
27	60–65	63	–	–	+	+	+
28	35–40					–	–
29	35–40					–	–
30	40–45					–	–
31	35–40					–	–
32	40–45					–	–
33	35–40	Epidemiological status: never travelled to the epidemic area and never were physically close to COVID-19 patients. COVID-19 symptoms: none.				–	–
34	25–30					–	–
35	30–35					–	–
36	45–50					–	–
37	35–40					–	–
38	45–50					–	–
39	30–35					–	–

a *Lung CT scans were taken every week before breath sampling, where all patients showed decreased glass shadows; swab PCR and routine blood tests were performed within 3 d before expiratory sampling; and antibody testing was performed within 7 d before expiratory sampling*.

“–”: no clinical symptoms/negative result; “+”: positive result; “*”: had two tests within 4 days apart but without a difference in results. The positive breath samples were further analysed by standard curve qPCR to determine the virus concentration (Nos. 25–27, shown in grey background).

The estimated number of virus particles in the three positive breath samples is shown in Figure 3 and Table 2. According to the RT-qPCR standard curve and original Ct data (Supplementary Table 1 and Supplementary Figure 1), the virus counts in RT-qPCR wells were 460, 171, and 255, while the ratio of virus copies in RT-qPCR wells after extraction and those in the original collection liquid after sampling was (200 μL/1.5 mL) × (10 μL/60 μL)=1/45. Thus, the total virus counts were estimated to be 20,700, 7,695, and 11,475 copies in each of the three positive breath samples. Considering the length of the breath collection process, which took 4.5, 4.5, and 5 min, the virus exhalations per minute were 4,600, 1,710, and 2,295 copies, respectively.

**Table 2:** Quantitative PCR analysis of SARS-CoV-2 in positive breath samples.

**Case #, sample type**	**Ct value**	**Copies in PCR well**	**Copies/min** **exhaled**
#25, Breath	35.00	460	4,600
#26, Breath	36.57	171	1,710
#27, Breath	35.94	255	2,295

## Discussion

To detect SARS-CoV-2 risk in air, we established a tool to collect exhaled human breath and detected nucleic acids of low concentrations of SARS-CoV-2 from patients with COVID-19. In this setting, no medical professional is needed, and other patients and scientists/healthcare workers are protected from exposure during the sampling procedure.

Our SAC device was able to detect SARS-CoV-2 RNA from air, and the detection limit of the device was obviously dependent on not only sampling time but also virus concentration. Based on the studies of sampling time-dependent tests with fixed virus concentration at 10,000 copies/mL and concentration-dependent tests with a fixed sampling time of half an hour, as shown in Figure 3, we found an explicit relationship for the threshold boundary with the product of virus source concentration and sampling time at approximately 42,000 s·copies/mL. When considering factors of the spraying rate of the sprayer, pumping rate of the collector, and distance dependent collection efficiency, we estimated the threshold boundary with the product of virus concentration in aerosol and sampling time at approximately 1.5 s·copies/mL, as shown in Supplementary Figure 2. Riediker and Tsai estimated concentrations in a room with an individual who was coughing frequently to be as high as 7.44 million copies/m^3^. However, regular breathing from an individual who was a high emitter was modelled to result in lower room concentrations of up to 1,248 copies/m^3^ (35). Indoor rooms with high air exchange rates up to 20 times per hour would be safe. However, concerning energy consumption and noise, strict ventilation is not the best choice. The current SAC-based method may provide a possibility to optimize the air exchange rate based on the test result. From our pseudovirus experiments, there was an 80-cm distance limitation for 1,800-s sampling, which was probably due to continuous air exchange in the biosafety cabinet, which stopped aerosol accumulation. Systematically controlled spraying in closed space is definitely important for a better understanding of breath transmission across aerosols, but it is beyond the emphasis of the current paper, showing the proof of principle of SAC for low-concentration virus detection from breath.

It is interesting to compare SAC with two other mainstream methods for collecting exhaled breath: condensation using a cooling device for aerosols with higher water content (EBC) and filtering that needs a high-efficiency particulate air (HEPA) filter or gelatine to transfer particles. SAC is favorable because of its high selectivity for virus particles (12, 13). Since SAC completes enrichment and dissolution of virus particles simultaneously, it has the potential to be integrated with a microfluidic system and subsequent PCR, in which automatic continuous detection of virus load becomes possible. Furthermore, SAC has been successfully employed to detect influenza virus (12, 13). Based on these facts, we hypothesized that it might be feasible to detect SARS-CoV-2 in the breath of patients via SAC. Exhaled droplets with a radius larger than 25 μm contain most of the viral load, following a parabolic trajectory onto surfaces where they settle and contaminate. For droplets with an initial radius between 2.5 and 25 μm, the dynamics are strongly dependent on Stokes law, with drag force from viscosity (36). Nevertheless, for aerosols with sizes below 2.5 μm that are hardly stopped by a surgical mask, gravity has a negligible effect unless they are in an environment with brackish air. Although the SARS-CoV-2 viral load in aerosol particles below 2.5 μm needs further clarification, current SAC devices are simulated to cover such a range down to 100 nm. Based on fluid dynamics analysis, two different groups independently revealed that the lifetime of a 10-μm droplet is approximately eight times longer when the relative humidity rises from 10 to 90% (37, 38), which might limit the quantitative analysis capability of EBC. However, SAC devices have no such influence from the environment because they are based on humidity-independent aerosol sampling.

Detecting virus content in breath is challenging, especially when the release concentration decreases and the sampling time is limited. Eiche and Kuster estimated the viral concentration in the exhaled liquid from 10^6^ to 10^11^/mL (39) using 5-min EBC, Ma et al. reported that earlier stage COVID-19 patients could exhale millions of SARS-CoV-2 particles per hour (23). The current research results show that later-stage COVID-19 patients could exhale SARS-CoV-2 at below 7,000 copies/min, corresponding to less than one copy per milliliter when considering an average exhale volume of approximately 7,000 mL/min (40). The detailed estimated data of virus content in clinical exhalations are shown in Supplementary Figure 2, with comparison to laboratory test data. If the SAC had been applied in an earlier stage where the release rate was approximately 90 times that of our result, as reported by Ma et al. (23), the sampling time would be reduced to <10 s. Moreover, in particular situations, such as with severely infectious individuals, showing the lowest swab Ct value of 19 (41), one-blow SAC sampling might be sufficient for fast screening.

There are limitations in this study, including the fact that only 3 of 27 subjects tested positive for SARS-CoV-2 by breath analysis. Notably, patients were in a late course of the disease. This result was maintained even after 9 of the 24 subjects underwent a second analysis. Viral load has been shown to be high in COVID-19 patients during the first week of symptoms and decrease during the second week (42, 43). Since most of the patients were asymptomatic at the time of sampling and had shown a long course of disease (23–70 d) prior to sampling, a low viral load is the most likely reason for their negative test results. To increase the positive rate for the SAC device, preclinical tests using pseudoviruses have shown that the suitable product of virus source concentration and sampling time is larger than the threshold 100,000 s·copies/mL, or the product of virus concentration in aerosols and sampling time is larger than the threshold 1.5 s·copies/mL. In principle, through very sophisticated clinical trials for patients, i.e., longer and higher frequency exhalation tests, it is feasible to determine whether the exhalation results are truly negative. Actually, clinical tests with a relatively long time, such as half an hour for patients, would not be necessary and practical for this study. Moreover, it is not possible to test additional patients in China at this time due to a shortage of patients with COVID-19. Nevertheless, improvements to the new method and systematic studies of influenza viruses are still in progress, and additional researchers are getting involved to push the method forward.

In short, breath testing with SAC for particle enrichment is a non-invasive and effective way to detect respiratory pathogens. By collecting and analysing the breath of COVID-19 patients, SARS-CoV-2 was identified in our study. Moreover, based on quantitative analysis of positive cases and estimation from the average tidal volume of adults, the virus in 5-min exhalations of the patients is detectable even when the virus concentration is less than one copy/mL. This study validated the concept that detection of SARS-CoV-2 in the breath of COVID-19 patients is feasible, implying potential applications for rapid screening of infectious individuals and automatic early epidemic prevention of respiratory pathogens in public environments.

## Data Availability Statement

The raw data supporting the conclusions of this article will be made available by the authors without undue reservation.

## Ethics Statement

The studies involving human participants were reviewed and approved by Peking University Third Hospital. The patients/participants provided their written informed consent to participate in this study.

## Author Contributions

XL was the principal clinical investigator. JLi was the principal investigator of laboratory evaluation. QG was an associate clinical investigator. YD was the principal investigator for nucleic acid testing kits. GL participated in the laboratory tests and discussion. WD participated in data analysis and discussion. WLi provided independent evaluation tests for laboratory evaluation. TZ conducted clinical tests in YouAn Hospital. LT analysed the fluid field of the swirling aerosol collection device. RZ performed SAC simulations in the different modes. XY performed clinical tests in TongJi Hospital. HZ participated in the laboratory tests. CZ participated in nucleic acid tests. WLiu contributed to pathogen analysis of expired viruses. LS produced the mechanical design for the collection device. KC organized the Lab and POC tests. ZW was the organizer of nucleic acid testing. NS organized the clinical researchers. JLu initiated the entire research project described in this paper. All authors contributed to the article and approved the submitted version.

## Conflict of Interest

The authors declare that the research was conducted in the absence of any commercial or financial relationships that could be construed as a potential conflict of interest.
