# Supplementary figures and images for: Detecting SARS-CoV-2 in the Breath of COVID-19 Patients
Source: Front Med (Lausanne). 2021 Mar 17;8:604392. doi: 10.3389/fmed.2021.604392 (PMC8010128; doi:10.3389/fmed.2021.604392)

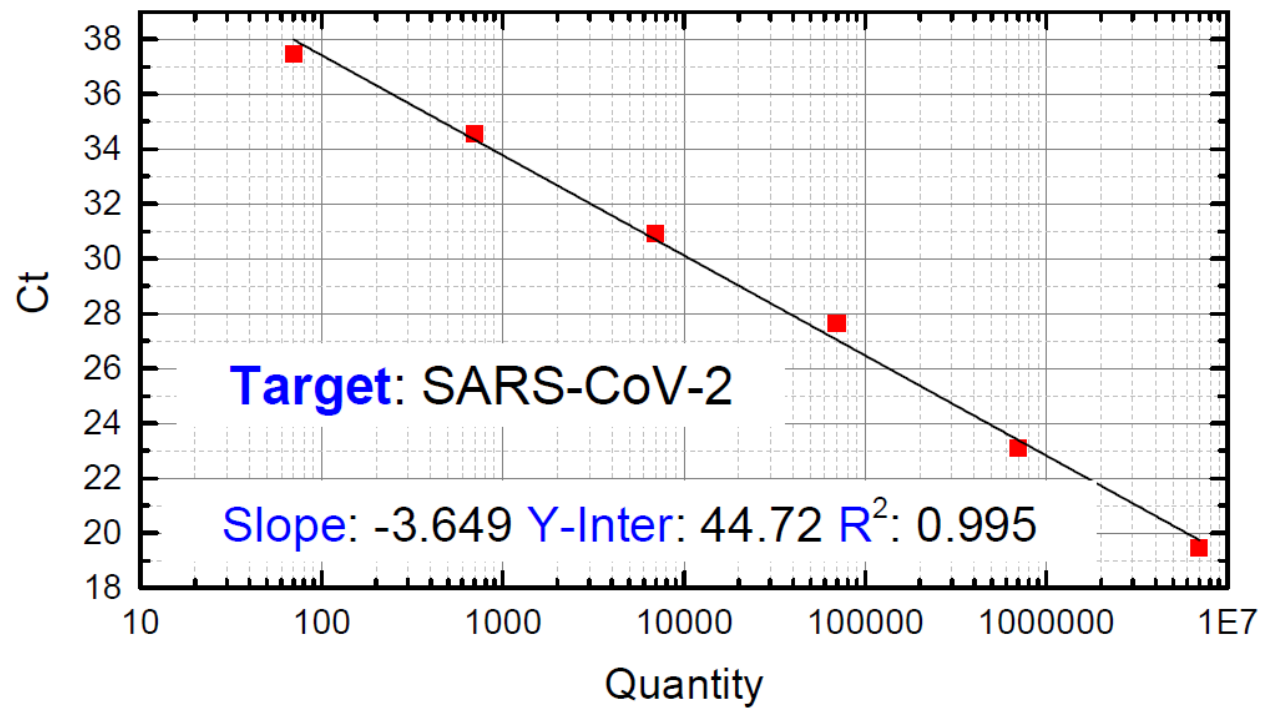

Supplementary Figure 1. Standard curve of the RT-PCR test for three positive SARS-CoV-2 cases.

Supplement: Supplementary file 1 [file Image_1.pdf]
