# Supplementary material for: Detecting SARS-CoV-2 in the Breath of COVID-19 Patients
Source: Front Med (Lausanne). 2021 Mar 17;8:604392. doi: 10.3389/fmed.2021.604392 (PMC8010128; doi:10.3389/fmed.2021.604392)
Supplement: Supplementary file 2 [file Image_2.pdf]

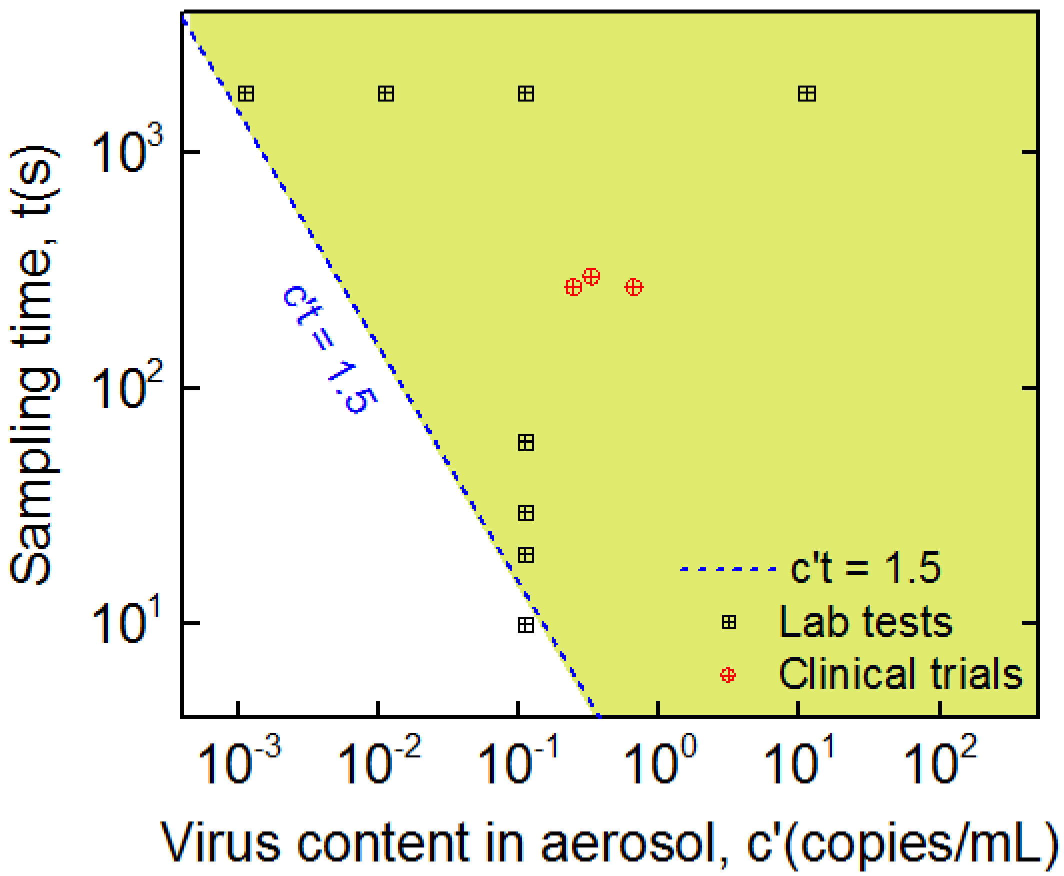

Supplementary Figure 2. Plot of sampling time versus estimated virus content in aerosols of laboratory tests and clinical exhalations, where the shaded area indicates the detection range of the current method based on laboratory tests, with the equation of the threshold boundary line c’t=1.5 (s·copies/mL). Here, the virus content in aerosol of Lab samples c’ is estimated using c’=cγα=c x [(100 mL/h)/(15 L/min)] x 0.1 = 0.000011c, where c is the concentration of pseudovirus solution, γ is ratio between spraying rate and pumping rate, and α is ratio of collection efficiency between distance of 10 cm and 0 cm derived from qPCR, while virus content in clinical exhalations is estimated referring to the average tidal volume of approximately 7,000 mL/min.
