# Supplementary material for: Detecting SARS-CoV-2 in the Breath of COVID-19 Patients
Source: Front Med (Lausanne). 2021 Mar 17;8:604392. doi: 10.3389/fmed.2021.604392 (PMC8010128; doi:10.3389/fmed.2021.604392)
Supplement: Supplementary file 3 [file Table_1.docx]

Supplementary Table 1. Raw RT-PCR test data for three positive COVID-19 breath samples.

| Well | Sample name | Target name | Ct | Quantity  (copies in PCR assay) |
| --- | --- | --- | --- | --- |
| A3 | # 25 | SARS-CoV-2 | 35.004795 | 459.652 |
| B3 | # 26 | SARS-CoV-2 | 36.574318 | 170.729 |
| C3 | # 27 | SARS-CoV-2 | 35.940468 | 254.698 |
| C7 | ST 6 | SARS-CoV-2 | 19.461578 | 7000000 |
| B7 | ST 5 | SARS-CoV-2 | 23.096119 | 700000 |
| A7 | ST 4 | SARS-CoV-2 | 27.656776 | 70000 |
| D7 | ST 3 | SARS-CoV-2 | 30.9326 | 7000 |
| E7 | ST 2 | SARS-CoV-2 | 34.548382 | 700 |
| B12 | ST 1 | SARS-CoV-2 | 37.481182 | 70 |
| H5 | P | SARS-CoV-2 | 26.861151 | 78374.758 |
| A5 | N | SARS-CoV-2 | Undetermined | Undetermined |

Note: A5 was the negative (N) control. H5 was the positive (P) control. A7, B7, B12, C7, D7 and E7 were the standards with known concentrations (STs). A3, B3, and C3 were the corresponding positive breath samples of patients #25, #26, and #27, respectively.
